# Haplotypes of SNPs associated with COX-2 and their comparison with histopathological features of breast cancer patients

**DOI:** 10.1186/2051-1426-3-S1-P9

**Published:** 2015-08-14

**Authors:** Muniba Aban, Imran Siddqui, Muhammad Saboor, Shahid Pervez, Tariq Moatter

**Affiliations:** 1 Pathology, Baqai Medical University, Karachi, Pakistan; 2 Molecular Pathology, Aga khan University and Hospital, Karachi, Pakistan

## Introduction

In breast cancer, increased cyclooxygenase-2 expressions is associated with poor prognosis. COX-2 expression influences production of pro-inflammatory prostaglandins. The present study investigated association between COX-2 promoter polymorphisms (rs689465, rs689466, rs20417) and histopathological features of breast cancer patients.

## Methods

We selected 150 HER-2 amplification positive breast cancer patients from our previous case-control study. The participants were evaluated for histopathological features and genotyped for COX-2 SNPs. Comparisons of genotype data with histopathological characteristics were performed by chi square test. Logistic regression was applied for estimation of odd ratios. COX-2 protein level and other markers were assessed by immunohistochemical staining. Statistical analyses were performed using SPSS version 19.0 and p value was set at <0.05.

## Results

Our data showed that elevated COX-2 expression was significantly associated with HER-2 amplified tumours. In addition, a positive association between rs20417 (GC+CC) and estrogens receptor (OR: 0.383, 0.161-0.913, P: 0.030) and IDC tumour (OR: 0.264, 0.070-0.993, P: 0.049) was noted. Eight haplotypes were deduced and associations with tumour size (P: 0.030), HER-2 amplification (P: < 0.0001), ER positivity (P: 0.017) were observed.

## Conclusion

Present study suggests that COX-2 expression and haplotypes of its associated SNPs should be considered for characterizing breast cancer prognosis.

